# The Need for Status, Organizational Citizenship Behavior, and Overall Evaluation: Focusing on the Moderating Effects of Political Skill and Task Visibility

**DOI:** 10.3390/bs14020105

**Published:** 2024-01-31

**Authors:** Jisung Park, Heesun Chae

**Affiliations:** 1 College of Business Administration, Chungnam National University, Daejeon 34134, Republic of Korea; jspark1@cnu.ac.kr; 2 School of Business Administration, Pukyong National University, Busan 48513, Republic of Korea

**Keywords:** the need for status, organizational citizenship behavior, overall evaluation, political skill, task visibility

## Abstract

Drawing on the costly signaling theory (CST), this study examined the need for status as a hidden motive to increase organizational citizenship behavior (OCB) and result in the focal employee’s overall evaluation. Moreover, as the activating cues, this study considered political skill as an individual characteristic and task visibility as a situational factor in the relationship between the need for status, OCB, and overall evaluation. To test these predictions, we obtained and analyzed 299 questionnaire responses from pairs of subordinates and supervisors in various South Korean industries. The analytical results supported our hypotheses that the need for status increased OCB, resulting in high performance appraisal. Moreover, task visibility positively moderated the relationship between the need for status and overall evaluation, which was mediated by OCB. However, political skill exhibited a negative moderation effect on this mediation process. These findings have important theoretical and practical implications, and they also highlight directions for future research.

## 1. Introduction

Organizational citizenship behavior (OCB) plays a critical role in enhancing organizational effectiveness [1,2]. Despite OCB being “not directly or explicitly recognized by the formal reward system” [2] (p. 4), such discretionary behavior is a desirable and recommendable virtue for employees and organizations [3,4,5]. However, despite the definite benefits of OCB from an organizational perspective [1], employees may face certain challenges regarding resource inputs and return uncertainties [5]. Specifically, although engaging in organizationally desirable behavior involves a loss of resources, an employee’s additional input of resource does not guarantee returns such as financial incentives and promotions [2]. In addition, it is impossible to guarantee that other employees will exhibit comparable behavior, even if one employee engages in OCB. In this regard, it is rational for an individual employee to devote all available resources toward fulfilling the duties and responsibilities (in-role behavior) prescribed in the job description. This is because doing so enhances the possibilities of high in-role performance, thereby resulting in formal rewards and promotions within the organization.

Despite these dilemmas, numerous employees still readily and voluntarily engage in OCB. Although self-sacrificial motives cannot fully explain this voluntary participation in OCB, accumulated studies have investigated other hidden motives and needs underlying these sacrificing behaviors [6]. For example, according to Rioux and Penner [7], OCB may arise from three motivations: organizational concern, prosocial values, and impression management. Unlike the first two motivations, impression management emanates from self-enhancement motives and utilizes rational human beings’ so-called OCB tactics to seek self-interest [8,9]. These OCB tactics can maximize returns by fostering favorable impressions with supervisors and colleagues. Consistent with these arguments, Bolino et al. [4] empirically demonstrated that supervisor-focused impression management tactics positively affect OCB ratings. In addition, there exists a positive relationship between citizenship behaviors and supervisor approval of the employee and overall job performance ratings. This favorable impact of an employee’s OCB extends to colleagues’ evaluations [10]. While this tactic approach expands our understanding of seemingly self-sacrificing behaviors, the resource-oriented explanation is more critical because extra-role behavior requires additional resource inputs. Tactic-based studies on OCB indicate that the retrieval of these resource investments is not constantly possible. In line with the functional perspective, motivation solely rooted in the desire for specific rewards is not sustainable, as the assurance of continuous and satisfactory rewards cannot be guaranteed [11]. Given these circumstances, a reexamination of the underlying motivations propelling employees to willingly engage in OCB becomes imperative. However, the current approaches to self-focused OCB have limitations in elucidating the fundamental cause of seemingly self-sacrificing behavior and its associated outcomes, primarily emphasizing short-term tactics [12,13]. To address these limitations, this study endeavors to unveil the latent motives within the subconscious and comprehensively explain the entire process of behavior–reward dynamics.

In this respect, based on studies on impression management and OCB, this study posits that the need for status within the organizational hierarchy is the hidden motive of impression management for conducting OCB [8,9]. In particular, the need for status is a deeper motive that explains why people willingly engage in extra-role behaviors such as knowledge sharing [14]. Moreover, this seemingly self-sacrificing behavior can result in rewards such as a higher evaluation from a supervisor or a positive reputation among colleagues [15]. In this aspect, examining the relationships among the need for status, OCB, and overall evaluation can provide a novel approach for understanding the reason and the outcome of OCB for the existing research field. To enhance the logical connections among variables, this study adopts costly signaling theory (CST). CST is a well-established framework that is utilized across various academic disciplines, including biology, management, sociology, and anthropology, to elucidate the underlying motivations behind OCB [15]. According to CST, individuals who prioritize making contributions to groups or organizations, even it means incurring substantial costs, rather than solely safeguarding their interests, ultimately regain the cost of their high-cost display through increased social prestige or recognition within the community [16,17,18]. By applying this concept to OCB, we propose that it functions as a costly signal for achieving reputation and compensation in the workplace. In this regard, OCB can signal the focal employee’s dedication and superiority. Moreover, given that the effectiveness of signaling can vary depending on individual and situational characteristics [14,19,20], this study utilizes political skill as an individual attribute and task visibility as a situational factor. This is because tacit skills and task characteristics can further activate the potentially hidden motive of the need for status. Specifically, political skills denote the capacity to adeptly discern other people’s needs and contextual dynamics, as well as to efficiently access the requisite resources from individuals or organizations to accomplish specific objectives [19]. These skills represent a personal competency that influences the selection of individuals and how commitment is demonstrated. Consequently, they serve as a facilitator, enabling individuals who have a pronounced need for status to effectively convey signals through OCB. Furthermore, in roles where one’s actions and contributions are apparent to a supervisor, amplifying one’s efforts and contributions heightens the impact of signaling through OCB; this is especially true for individuals with a strong need for status.

**Figure 1:**
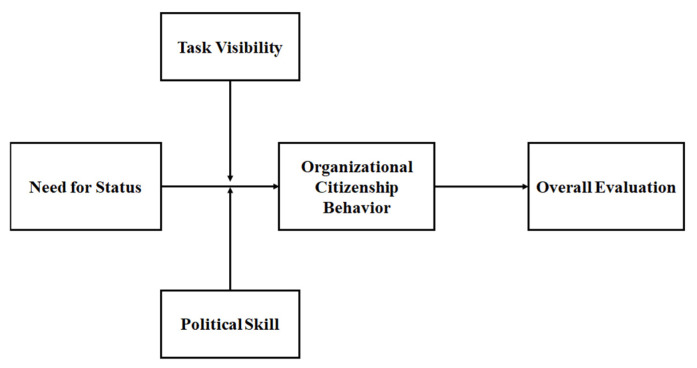
Research model.

In summary, we investigate the hidden motive (the need for status) for OCB and ultimate returns (overall evaluation) by setting an individual’s political skill and task visibility as moderating variables. Specifically, we examine the main arguments of the costly signaling theory. Next, we hypothesize the effect of the need for status on OCB and the resultant overall evaluation by a leader, as well as the moderating effects of political skill and task visibility in the relationships between the need for status, OCB, and overall evaluation. To empirically test the theoretical propositions, we use multi-source data, including supervisor–subordinate dyads collected from South Korea organizations. Figure 1 shows the detailed research model. 

## 2. Theoretical Background and Hypotheses

### 2.1. Costly Signaling Theory

The biological field first proposed CST to explain why animals, including human beings, voluntarily share their valuable resources with others [18]. Between the early 1960s and 1970s, there was considerable focus on kin selection, which emphasizes sharing among agents with close DNA or reciprocity that stresses induce [15]. These arguments on why agents share their resources with others are sound; however, they still have fundamental limitations, such as the inability to account for un-reciprocal offerings among non-related agents. CST is proposed as an alternative explanation to investigate the hidden motive for resource sharing as a way for individuals to signal their hidden qualities [18,20]. According to CST, the seemingly self-sacrificial behavior may stem from the need for status to enhance reputation within organizational hierarchies by investing additional resources into public affairs [15,16,17,18,20]. In other words, from a short-term perspective, investing valuable resources for the common good of a group or a community appears to be a meaningless waste of resources. However, such sacrificial behavior ultimately compensates for short-term disadvantages with long-term benefits, such as competent partners or superior mates. An individual’s unselfish contribution to the community enhances their reputation by demonstrating one’s generosity and capability and may result in compensation such as a pay raise or promotion. In this regard, Zahavi [18] stated that “prestige may be gained by investing in wasteful characters [behaviors] as well as in altruistic activities” (p. 2). 

Consistent with these arguments, numerous studies on CST have demonstrated that it is an effective strategy to invest excessive resources on the community. For example, Barclay [16] and Smith and Bliege Bird [17] showed that throwing a housewarming party or sharing food were interpreted as signals of altruism and generosity, which resulted in monetary returns from the group. Hardy and Van Vugt [21] and McAndrew [22] reported that others highly evaluate people who behave altruistically within communities. Similarly, Gintis et al. [20] indicated that, from an evolutionary perspective, signaling by self-sacrificing for others is the most robust strategy. In addition, Park et al. [14] stated that voluntary knowledge sharing is an effective form of signaling for enhancing reputation and evaluation.

### 2.2. Need for Status, Organizational Citizenship Behavior, and Overall Evaluation

As previously noted, OCB is an organizationally desirable behavior that enhances the overall functioning of the organization [1,2]; however, it is not directly or explicitly recognized by the formal reward system [2]. It implies that even if employees are actively involved in OCB, they cannot be guaranteed any rewards from an organization. In this regard, spending all resources to perform in-role behavior is the best strategy for an individual employee from a rational decision-making perspective. Such a strategy increases the possibility of high in-role performance and the resulting rewards [6]. Of course, this OCB may emanate from pure organizational concern or prosocial values [7]. Moreover, OCB has been reported to have positive relationships with the supervisor’s liking of the employee and overall job performance rating [4,10]. Nevertheless, these explanations cannot correctly justify an alternative motive for OCB participation. 

As mentioned above, CST proposed that employees willingly share their extra resources, such as time and effort, because such behavior enhances their reputation and social prestige by demonstrating their superior qualities and passionate dedication to the collective [18,20]. Their abilities will be constrained if they can only perform in-role behaviors because they lack additional resources. In addition, if they are uninterested in an organization’s overall functioning and sustainability, they perform the duties and responsibilities outlined in their job descriptions. In this aspect, conducting OCB implies the focal employee’s superiority and commitment to the organization. Moreover, others will highly recognize this voluntarily desirable behavior, especially supervisors with formal authority to evaluate team members [14].

However, the frequency of actual OCB may vary depending on the degree of personal motive [10]. Previous studies have revealed that individuals with strong upward desires tend to flaunt their abilities at an enormous cost [21,22]. In this manner, compared with employees with a low need for status, those with a high need for status have a strong desire to attain organizational status and prestige [14]. They will participate in OCB more frequently because OCB is an effective signal that highlights these employees’ superiority and dedication within the organization. In this regard, Park et al. [14] asserted that “the need for status can be the most powerful and fundamental motive to elicit socially desirable behavior” (p. 24). Therefore, we predict that the need for status will increase OCB. In addition, proactive engagement in OCB can enhance supervisors’ recognition and resultant overall evaluations because, as organizational agents, supervisors place a greater emphasis on contributions to the collective [23]. Previous studies also demonstrated that the supervisor or colleagues reward organizationally desirable behavior through high evaluation, status, or reputation within an organization. [14,21,22]. Therefore, OCB will function as an intermediate means to achieve organizational recognition and social hierarchy, which individuals with a high need for status pursue.

**Hypothesis** **1:** *OCB mediates the relationship between the need for status and overall evaluation.*

### 2.3. Moderating Role of Political Skill

Individual characteristics and contextual factors interact to yield outcomes based on perceptions. An individual’s tacit skills can further activate the potentially hidden motive of the need for status. As a typical tacit skill, political skill can be defined as “the ability to effectively understand others at work and to use such knowledge to influence others to act in ways that enhance one’s personal and/or organizational objectives” [24] (p. 127). Based on the earlier definition, Ferris et al. [19] defined political skill as “a comprehensive pattern of social competencies, with cognitive, affective, and behavioral manifestations, which have both direct effects on outcomes as well as moderating effects on predictor–outcome relationships” (p. 291). This political skill comprises four interrelated dimensions: apparent sincerity, social astuteness, interpersonal influence, and networking ability. Based on these four dimensions’ characteristics, politically skilled individuals are evaluated as reliable, clever, attractive, and extensively networked. Consequently, they can access information concerning their work environment and secure the required resources. Consistent with these characteristics, previous studies on political skill have revealed that it is positively associated with task performance, OCB, contextual performance, evaluation, personal reputation, and career success [25,26]. Moreover, in light of recent research findings, it has been identified that political skill can mitigate adverse occurrences in the workplace. For instance, a heightened level of political skill has been associated with a reduced likelihood of employees experiencing workplace cyberbullying [11] and has been shown to alleviate negative perceptions of organizational politics [12]. This is attributed to the political skill facilitating greater access to resources and fostering diverse networks within the organization.

As previously mentioned, because an employee’s strong need for high status is a form of subconscious motive, some individual skills can stimulate such a hidden motive. Political skill is the ability to effectively capture others’ needs and environmental conditions and efficiently obtain the necessary resource pools from others or an organization [19,23], thereby achieving one’s intended goals. In this regard, good political skills can amplify the effect of the need for status on OCB by increasing the possibility of attaining social prestige and reputation. In contrast, the impact of such desire on OCB will not be substantially high in the case of people with low political skills because the individual characteristic’s cue to activate the need for status is somewhat weak. Therefore, political skill will moderate the relationship between the need for status and overall evaluation as mediated by OCB. Therefore, we propose the following moderated mediation hypothesis.

**Hypothesis** **2:** *Political skill moderates the relationship between the need for status and overall evaluation as mediated by OCB, such that the mediated relationship is stronger when political skill is high than when it is low*.

### 2.4. Moderating Role of Task Visibility

Task visibility is defined as “an employee’s belief that a supervisor is aware of the employee’s individual effort on the job” [27] (p. 446). This job characteristic influences an individual’s judgment and interpretation of resource inputs when performing the task. According to the social loafing literature, in a situation where people can easily hide in the crowd, they are likely to withdraw their resource inputs, including time and effort, based on rational decision making [20,28,29]. In other words, when people perceive outcomes as inseparable from collective contributions, withholding efforts is the best strategy from each individual’s rationality perspective. On the contrary, when leaders can effectively monitor each follower’s separate effort and independent performance, followers are likely to complete unassigned duties and responsibilities to achieve a high overall appraisal and social reputation [29,30].

As previously mentioned, based on the situation, the broadcast effectiveness of costly signaling can markedly vary [14]. High task visibility creates an effective environment where employees can appeal to their efforts and contributions to the organization. They can do this by engaging in organizationally desirable behaviors [20,27], considering that a supervisor, as an organization agent, makes a final decision on the employee evaluation [22]. In other words, compared to the situation with low task visibility, employees can easily reveal their prosocial behaviors for the common good under high task visibility. As a result, these friendly behaviors will be highly recouped by a high evaluation, including a pay raise and promotion. In this regard, this situation will facilitate the impact of the need for status on OCB because high task visibility can compensate for resource-wasting effects for the collective by maximizing the effectiveness of costly signaling. Therefore, we propose the following hypothesis.

**Hypothesis** **3:** *Task visibility moderates the relationship between the need for status and overall evaluation as mediated by OCB, such that the mediated relationship is stronger when task visibility is high than when it is low.*

## 3. Methods

### 3.1. Sample and Procedures

To validate the hypotheses proposed in this study, we conducted a targeted survey focusing on major corporations situated in the Seoul metropolitan area and the Gyeonggi region of South Korea. This selection specifically included a diverse range of industries such as electronics, telecommunications, automotive, and manufacturing, ensuring a comprehensive representation of key sectors in these regions. In our effort to enhance the robustness of our research and address potential common method biases [31], we implemented a dual-structured survey approach. This entailed crafting two distinct questionnaires—one for employees and another for their immediate supervisors. The process of selecting companies for our study was executed with meticulous attention, concentrating on prominent businesses in Seoul and the Gyeonggi region. We proactively engaged executives of these corporations through well-established professional networks and collaborations with industry associations. Leveraging these connections, we formally requested their participation in our data collection process, explicitly emphasizing the study’s exclusive academic nature. Importantly, all participants voluntarily engaged in the survey, having been duly informed that the collected data would be used solely for academic purposes.

A total of 330 survey questionnaires were distributed, of which 305 were successfully retrieved, yielding a response rate of 92.4%. Post screening, 299 surveys were considered pertinent for the ultimate analysis, discounting 6 due to apparent response irregularities or incongruities in supervisor–subordinate matches.

The examination of the demographic characteristics of the respondents employed in the final analysis revealed that the average age of employees was 35.2 years and the average organizational tenure of employees was 5.89 years, with 153 male respondents accounting for 51.2% of the total. In terms of educational attainment, 4 respondents (1.3%) had a high school degree, 79 (26.4%) held a two-year vocational college degree, 185 (61.9%) had a university degree, and 31 (10.4%) possessed postgraduate qualifications.

### 3.2. Measures

The scales of the survey items used in this paper were adopted from prior studies that have established their validity and reliability. With the exception of demographic variables, all items were constructed using a 7-point Likert scale (1 = Not at all, 7 = Very much).

***Need for status.*** The eight survey items (α = 0.92) developed by Flynn et al. [32] were utilized to gauge the extent of meaningful respect and recognition within colleagues or respective groups. Examples of survey items include ‘I want my peers to respect me and hold me in high esteem’, and ‘I enjoy having influence over other people’s decision making’.

***Political skill.*** To assess political skill, a shortened eight-item (α = 0.90) version of the Political Skill Inventory (PSI; Ferris et al. [24]) was employed. The PSI comprises four dimensions: networking ability, social astuteness, interpersonal influence, and apparent sincerity. Two items from each dimension were chosen based on their highest loading in preceding factor analyses. Examples of survey items include ‘I always seem to instinctively know the right thing to say or do to influence others’, and ‘At work, I know a lot of important people and I am well connected’.

***Task visibility.*** The six survey items (α = 0.88) developed by Liden et al. [30] were employed to evaluate an employee’s belief that a supervisor is aware of the employee’s individual contribution and effort on the job. Examples of survey items include ‘My supervisor is aware of the amount of work I do’, and ‘My supervisor usually notices when an employee is slacking off’.

***Organizational Citizenship Behavior.*** The sixteen survey items (α = 0.96) developed by Lee and Allen [33] were utilized to gauge individual discretionary actions that foster the efficient functioning of the organization. These behaviors are not explicitly acknowledged by the organization’s formal compensation system and were evaluated by the immediate supervisor. Examples of survey items include ‘This employee helps others who have been absent’, and ‘Express loyalty toward the organization’.

***Overall evaluation***. The five survey items (α = 0.97) developed by Allen and Rush [34] were utilized to assess the perceived importance and value of a employee within the organization. This evaluation was conducted by the immediate supervisor, and examples of the specific questionnaire items include ‘This employee makes important contributions to the organization’, and ‘This employee would be extremely costly to replace’.

***Control variables.*** This study controlled for four demographic variables with the intention of enhancing the validity of the hypothesis testing results and excluding logically explicable alternative models [9]. Specifically, drawing on previous research, individual characteristics, namely, age, gender, organizational tenure, and educational level, were included as control variables. Gender was dichotomized using a nominal scale, with ‘male = 0’ and ‘female = 1’ being dummy-coded. Educational level was measured on an ordinal scale (high school graduate = 1, vocational college graduate = 2, university graduate = 3, postgraduate or higher = 4), while age and organizational tenure were measured in years. This was done to elevate the validity of the results of hypothesis testing and to preclude alternative models that could be logically explained.

### 3.3. Analysis Strategy

The SPSS 27 software was employed to validate this study’s hypotheses. Cronbach’s α values were utilized to assess the survey items’ reliability. Using AMOS 27, exploratory and confirmatory factor analyses were conducted to ascertain the model’s discriminant validity. Subsequently, a correlation analysis was conducted to determine the degree of correlation among variables. Hierarchical multiple regression analysis was used to validate the research model’s hypotheses. Additionally, mediating and moderated mediation effects were examined using the bootstrapping procedure proposed by Preacher et al. [35]. Furthermore, the approach proposed by Aiken and West [36] was adopted to delve deeper into moderation effects. By distinguishing between high and low values of one standard deviation from the mean of the moderator variable, interaction effects were visually presented. T-tests were conducted to ascertain each level’s significance of regression coefficients (simple slope).

## 4. Results

### 4.1. Reliability and Validity Verification

A preliminary exploratory factor analysis was conducted to establish the validity of the measurement instruments. Using principal component analysis with Varimax orthogonal rotation, the exploratory factor analysis retained only factors with eigenvalues greater than or equal to 1. Table 1 presents the results of the exploratory factor analysis, which yielded a six-factor solution. Consequently, it can be inferred that the five variables in this study are composed of conceptually independent constructs. Furthermore, all factor loadings exceeded 0.50, indicating satisfactory validity [37], and the cumulative variance explained was 67.48%, exceeding the 60% threshold.

To assess the internal consistency among survey items, Cronbach’s α coefficients were computed to evaluate reliability. Typically, a Cronbach’s α coefficient above 0.7 indicates satisfactory reliability [38]. Upon examining the Cronbach’s α values for each factor as presented in Table 1, it is evident that the variables exhibited a high level of reliability: 0.92 for ‘need for status’, 0.90 for ‘political skill’, 0.88 for ‘task visibility’, 0.96 for ‘OCB’, and 0.97 for ‘overall evaluation’.

### 4.2. Descriptive Statistics and Correlation Analysis

The confirmation of normal distribution for the data was ascertained based on the skewness and kurtosis values. As all values fell within the acceptable range of ±2 [39], they satisfied the assumption of normal distribution. As shown in Table 2, we performed the descriptive statistical analysis and correlation analysis to investigate the correlations between the major variables. Examining the results of the descriptive statistics and correlation analysis presented in Table 3, it is evident that the independent variable ‘need for status’ exhibited statistically significant positive correlations with the mediating variable ‘OCB’ (r = 0.32, *p* < 0.001) and the outcome variable ‘overall evaluation’ (r = 0.20, *p* < 0.01), as well as with the moderating variable ‘political skill’ (r = 0.49, *p* < 0.001). However, no significant correlation was observed between ‘need for status’ and the another moderating variable, ‘task visibility’ (r = 0.11, *ns.*). Furthermore, a high positive correlation was observed between the mediating variable ‘OCB’ and the outcome variable ‘overall evaluation’ (r = 0.76, *p* < 0.001).

### 4.3. Confirmatory Factor Analysis (CFA)

To validate the empirical distinctiveness of the variables in the present study, a confirmatory factor analysis was conducted. A model is deemed fit if its comparative fit index (CFI) and Tucker–Lewis index (TLI) are above 0.90, and its root mean square error of approximation (RMSEA) is below 0.10 [40]. Examining Table 3, the proposed five-factor structure model in this study demonstrated an overall sound fit with satisfactory fit indices (x^2^[821] = 1951.71, *p* < 0.001, CFI = 0.90, TLI = 0.90, RMSEA = 0.07). This indicates superior fit compared to alternative models with fewer than four factors. Based on these confirmatory factor analysis results, the hypotheses were tested using the variables ‘need for status’, ‘political skill’, ‘task visibility’, ‘OCB’, and ‘overall evaluation’.

### 4.4. Hypothesis Testing

In Hypothesis 1, we predicted that OCB would mediate the relationship between the need for status and overall evaluation. To validate the mediating effect hypothesis, we employed Baron and Kenny’s [41] step-by-step regression analysis. In the initial phase, it is necessary for the independent variable to have a statistically significant impact on the mediating variable. As evident in Model 4 of Table 1, the need for status exhibited a statistically significant relationship with OCB (Beta = 0.26, *p* < 0.001). Subsequently, the second stage demands the independent variable to wield a statistically significant effect on the dependent variable. Model 4 of Table 4 corroborates the statistically significant association between the need for status and overall evaluation (Beta = 0.16, *p* < 0.01). During the final step, once these prerequisites are fulfilled, the mediating variable validates its impact on the dependent variable, and the effect of the independent variable on the dependent variable should become statistically insignificant or diminish when both variables are simultaneously introduced. In such instances, a lack of significant influence signifies complete mediation, whereas a reduced significant effect denotes partial mediation. When OCB was added to Model 7 (compared to Model 4), the need for status was no longer statistically significant in relation to overall evaluation (Beta = −0.03, *ns*.), whereas the mediating variable, OCB, became statistically significant (Beta = 0.80, *p* < 0.001). This indicates that OCB completely mediates the relationship between the need for status and overall evaluation. The mediation analysis results through bootstrapping procedures [35] also confirmed the complete mediating effect of OCB (point estimate = 0.27, Sobel Z = 4.66, 95% confidence interval (CI) of 0.14 and 0.39). Therefore, Hypothesis 1 is supported.

Hypothesis 2 predicted that political skill would moderate the relationship between the need for status and overall evaluation as mediated by OCB. To test the moderated mediated effect, we followed the procedures proposed by Preacher et al. [35]. First, we examined whether the interaction between the need for status and political skill significantly predicted OCB. Second, we explored whether the indirect effect of OCB varied based on the level of political skill.

**Figure 2:**
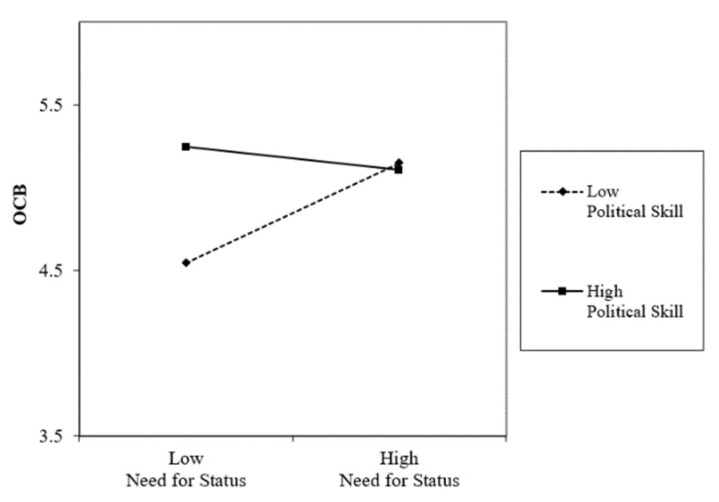
Interaction effects of need for status and political skill on OCB.

As shown in Model 3 of Table 4, political skill significantly moderated the relationship between the need for status and OCB (Beta = −0.18, *p* < 0.01). To delve deeper into political skill’s significant moderation effect graphically, we followed the procedures outlined by Aiken and West [36] to depict the interaction effect. As shown in Figure 2, the positive relationship between the need for status and OCB strengthens when political skill is low. In contrast, the effect of the need for status on OCB remains relatively constant when political skill is high.

Furthermore, bootstrapping procedures [35] confirmed the moderated mediated effect. As demonstrated in Table 5, the indirect mediating effect of OCB was significant when political skill was low (point estimate = 0.29, *p* < 0.01, 95% CI of 0.07 and 0.50) but not significant when it was high (point estimate = 0.09, *ns*., 95% CI of −0.05 and 0.33). Therefore, although the mediated moderation effect in Hypothesis 2 was confirmed, the direction of the moderation effect was contrary to the predicted hypothesis.

**Figure 3:**
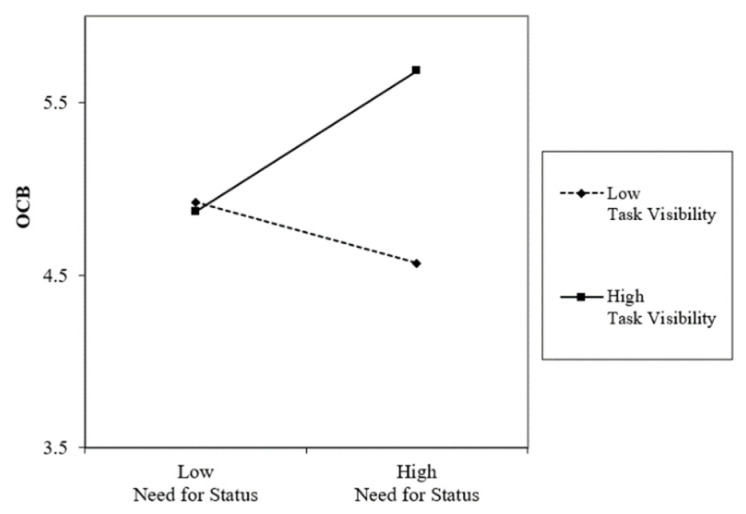
Interaction effects of need for status and task visibility on OCB.

In Hypothesis 3, we predicted that task visibility would moderate the relationship between the need for status and overall evaluation as mediated by OCB. As shown in Model 3 of Table 4, task visibility significantly moderated the relationship between the need for status and overall evaluation (Beta = 0.17, *p* < 0.01). Figure 3 illustrates that the positive relationship between the need for status and overall evaluation strengthens when task visibility is high. In contrast, no significant change was observed in the relationship between the need for status and overall evaluation when task visibility is low.

These results were also confirmed through bootstrapping procedures. Table 5 shows that the indirect mediating effect of OCB was significant when task visibility was high (point estimate = 0.26, *p* < 0.01, 95% CI of 0.13 and 0.41); however, it was not significant when task visibility was low (point estimate = 0.19, *ns*., 95% CI of −0.01 and 0.38). Therefore, Hypothesis 3 is supported.

## 5. Discussion

### 5.1. Overall Findings

By applying costly signaling theory, this study examined the hidden motives that drive individuals to willingly invest their effort and energy to engage in OCB behaviors. Specifically, it explained that OCB can serve as evidence of one’s effort and superiority and presented situational variables that can reinforce these motives. It considered individual characteristics, such as political skill, which allow individuals to concretize their efforts, provide exceptional interpretations of their surroundings, and engage in appropriate behavioral interventions and task visibility, which can enhance one’s perceived value during job performance.

These rationales were empirically analyzed using data collected from employees of South Korean companies in various industries. As predicted, the verification results revealed that individuals with a higher need for status engaged in OCB more actively, resulting in positive overall evaluations of the individuals. In particular, OCB entirely mediated the relationship between the need for status and employee evaluation. This indicates that self-oriented motives, such as the need for status, serve as a comprehensive catalyst for fostering altruistic outcomes from an organizational standpoint. This finding can be understood through the lens of the interplay between individual differences and national culture. As indicated in prior research, the motivations behind OCB can exhibit variations across countries, particularly between Asian and Western cultures [13,42]. Given that our data were collected from South Korean firms embedded in a collectivistic culture, it is crucial to contextualize the interpretation of our findings. Despite all employees being equally influenced by the collectivistic culture in South Korea, our study revealed differences among individuals. This discovery underscores the significance of showcasing self-superiority to enhance social status within the organizational hierarchy. However, even more crucial is the indirect revelation of one’s hidden competence through seemingly unselfish contributions to the community, strategically avoiding damage to interpersonal relationships. In a collectivistic culture, overt bragging may become a target of envy and bitterness, echoing the proverb, ‘An angular stone is bound to be hit by a chisel.’ Furthermore, considering power distance becomes essential, as the need for status may be more pronounced in collectivistic cultures, where power and rewards are relatively concentrated among high-status individuals within the community [43,44]. In such a scenario, if an employee harbors a strong need for status, they are likely to be more engaged in prosocial behaviors, including OCB. Particularly noteworthy is the role of signaling for employees when a supervisor, possessing the authority to evaluate them, perceives a high power distance. In this context, an employee’s actions, such as assisting colleagues and contributing to the overall organization, assume heightened importance as a tactic for impression management toward the supervisor.

Notably, contrary to what was predicted, political skill negatively moderated the relationship between the need for status, as mediated by OCB, and overall evaluation. On the contrary, as predicted, task visibility positively moderated it. The following are the theoretical and practical contributions resulting from these intriguing findings. Some explanations can be possible regarding the opposite result from the initial prediction. The relative strength of inborn needs compared to acquired learning, including political skill, is one possibility. In other words, when an individual has a strong need for status, such desire can be a powerful driver to induce seemingly self-sacrificing behavior like OCB in order to earn a higher social hierarchy. Because a strong need for status reduces the variations among individuals, a high level of political skill may no longer work as a simulator. On the contrary, if an individual’s need for status is low, the degree of OCB will be higher when he or she intentionally utilizes his or her political skill as an acquired skill set to supplement the lack of motivation (the need for status) for OCB.

### 5.2. Theoretical Implications

First, this study expands the theoretical explanation of why people voluntarily engage in OCB by drawing on costly signaling theory. Indeed, researchers such as Rioux and Penner [7] have suggested that diverse motivations, including organizational concern, prosocial values, and impression management, can trigger OCB. In certain aspects, these motivations can explain why people willingly participate in OCB. However, comprehension regarding the factors that stimulate seemingly sacrificing behaviors by allocating additional resources and how these prosocial behaviors ultimately yield long-term benefits for the group or community remains limited. To crystalize the triggers and mechanism, we adopted CST as a multidisciplinary, overarching theory [14]. Consequently, this study discovered that employees with a strong need for status were more likely to engage in OCB. Moreover, high evaluation by supervisors can compensate for this dedication to the organization. Consistent with prior research [4,5,6], this discovery affirms that an organization can provide rewards for engaging in behavior that benefits the organization, even if it is not explicitly specified in the job description. In this regard, CST can enhance our understanding by exploring why some individuals willingly share their valuable resources to engage in sacrificing behaviors that are not prescribed in the job description. Therefore, to reconcile the motivational dilemma between an individual’s altruistic choice and an organization’s necessity for effective functioning, this study expands the theoretical boundaries.

Second, our findings offer new insights into the factors that activate engagement in OCB regarding the broadcast effectiveness of signaling. To articulate these factors, we investigated a personal skill and a situational condition. This study examined the factors that influence the relationship between the need for status, OCB, and overall evaluation. It examined how personal and contextual factors impact people’s willingness to engage in OCB, considering that interactions between individuals and their environment influence human behavior.

The analytical findings demonstrate that task visibility acts as a situational cue to stimulate the need for status, in the sense that such a task characteristic makes prosocial behavior more pronounced to their supervisor and colleagues. Moreover, employees with a strong desire for social status and a high level of political skills are likelier to engage in OCB to display their “stand-out” needs through effective tactics. Even more intriguing is the fact that the impact of political skill on OCB indicates the most pronounced difference when the need for status is low. In other words, even when the need for status is low, a high level of OCB results from having the political skill to accurately assess the surrounding circumstances and employ effective behavioral strategies. These findings once more emphasize the significance of situational variables that stimulate individuals’ hidden motives to promote OCB. Thus, by providing more elaborate explanations for human behavior based on a hidden motive, these findings can broaden the current research streams in the OCB literature.

### 5.3. Managerial Implications

The implications of our findings are manifold for managers. First, leaders can use human resource management differentially based on individual needs and skills. This can be achieved by understanding how each employee perceives additional responsibilities as a burden or an opportunity. For example, subordinates with a high need for status view additional responsibilities as excellent opportunities for attaining high prestige and reputation within organizational hierarchies. In contrast, subordinates with a low need for status perceive them as a waste of resources. Second, organizations can contemplate designing effective job characteristics to enhance employees’ participation in OCB. As previously mentioned, numerous studies on social loafing have demonstrated that employees tend to withdraw their efforts to perform in-role and extra-role behaviors when task visibility is low [20,28,29]. Therefore, to increase the overall degree of employees’ OCB, organizations should provide more concrete information regarding jobs and establish more visible environments to promote easy monitoring by a supervisor. Third, organizations must re-design evaluation systems and effectively communicate the positive outcomes of conducting OCB to increase employees’ engagement.

### 5.4. Limitations and Future Research

Several limitations should be addressed in the discussion of the current findings. First, the analysis did not establish causality because cross-sectional data were utilized. To overcome this limitation, supervisor–subordinate dyad data sets were utilized in this study, which was also based on solid theoretical foundations. In other words, this study utilized CST as an overarching theory to predict causality when formulating hypotheses. Nevertheless, future studies should corroborate the causality among variables with longitudinal data.

Second, the potential effects of organizational culture or the compensation system on prosocial behavior within the organization were not considered in this study. Instead, this study focused on examining the variations in individual characteristics related to OCB. However, considering that the degree of individuals’ OCB can markedly differ depending on organizational culture (i.e., clan culture; [45]) and compensation systems (i.e., evaluation and reward systems; [46]), future research should adopt a multilevel design to examine these organizational characteristics that impact individuals’ OCB.

Third, as a boundary condition, this study solely focused on task visibility, which may have resulted in the exclusion of other task characteristics that could have an impact on the relationship between the need for status and OCB. For instance, task significance can be crucial in activating prosocial behaviors such as knowledge sharing and voice behavior. Therefore, to develop a comprehensive and contextualized understanding of OCB based on CST, additional predictors and contingencies, such as individual differences, interpersonal connectivity, and other contextual factors [2,5], need to be explored. Such research will enhance the value and implications of this study’s findings.

Fourth, although this study considered OCB at the individual level, the concept of OCB can be investigated at the group level [47]. The hidden motives individuals perceive when engaging in OCB may vary in groups with high OCB levels. Additionally, it may be necessary to exercise discretion regarding whether and to what extent individuals should engage in OCB in groups with low OCB levels. In such instances, the moderating role of an individual’s political skills may be emphasized. Therefore, going beyond the individual level of OCB and considering political influence based on the relative OCB level in team dynamics (perceived OCB gap or OCB difference) could provide novel and broader research avenues.

## 6. Conclusions

Human behavior is influenced by intricate motivations that are interwoven with both selfish and altruistic desires. In addition, despite the selfish motive, the behavior may exhibit altruistic qualities that benefit both the individual and the organization. This puzzling phenomenon is the subject of this study, and we believe that CST can provide an insightful perspective on employees’ additional resource inputs through behaviors that conspicuously benefit organizations and, ultimately, enhance their social reputation and standing. This study empirically demonstrated that such seemingly self-sacrificing behavior could be compensated by a rise in social standing (high employee evaluation by supervisors). We hope that this study’s findings, notwithstanding its limitations, will inspire other researchers to further investigate the covert incentives behind facilitating OCB.

## Figures and Tables

**Table 1:** Exploratory factor analysis and reliability analysis.

Items	Factor 1 (OE)	Factor 2 (PS)	Factor 3 (NS)	Factor 4 (TV)	Factor 5 (OCB)	Cronbach’s Alpha
OE1	**0.882**	−0.010	0.097	−0.027	0.062	0.97
OE2	**0.896**	−0.010	0.067	−0.064	0.019	
OE3	**0.910**	−0.043	0.093	−0.051	0.053	
OE4	**0.863**	−0.023	0.105	−0.030	−0.015	
OE5	**0.871**	−0.003	0.074	−0.026	0.056	
PS1	0.184	**0.600**	0.169	0.210	−0.052	0.90
PS2	0.072	**0.782**	0.138	0.124	0.046	
PS3	0.041	**0.813**	0.150	0.199	0.067	
PS4	−0.020	**0.803**	0.128	0.061	0.166	
PS5	0.100	**0.779**	0.207	0.072	0.185	
PS6	0.161	**0.774**	0.203	0.034	0.092	
PS7	0.115	**0.615**	0.240	0.016	0.080	
PS8	0.103	**0.671**	0.230	−0.012	−0.026	
NS1	0.137	0.245	**0.737**	0.036	0.033	0.92
NS2	0.222	0.382	**0.670**	0.090	0.042	
NS3	0.183	0.188	**0.791**	0.059	−0.089	
NS4	0.226	0.217	**0.755**	−0.013	−0.065	
NS5	0.154	0.214	**0.817**	−0.051	−0.021	
NS6	−0.046	0.183	**0.749**	0.036	0.211	
NS7	−0.040	0.082	**0.781**	0.013	0.214	
NS8	−0.035	0.141	**0.716**	0.041	0.263	
TV1	0.040	0.246	−0.031	**0.650**	−0.034	0.88
TV2	0.060	0.077	−0.030	**0.754**	0.069	
TV3	−0.025	0.099	0.007	**0.860**	0.091	
TV4	0.115	0.122	0.028	**0.751**	−0.083	
TV5	0.052	0.015	0.091	**0.875**	0.123	
TV6	0.116	−0.004	0.085	**0.791**	0.066	
OCBI1	0.354	0.062	0.169	0.033	**0.696**	0.96
OCBI2	0.384	0.105	0.140	0.029	**0.640**	
OCBI3	0.316	0.144	0.079	0.097	**0.640**	
OCBI4	0.322	0.166	0.196	0.038	**0.679**	
OCBI5	0.328	0.061	0.117	0.145	**0.658**	
OCBI6	0.300	0.227	0.133	−0.006	**0.640**	
OCBI7	0.375	0.173	0.156	0.076	**0.566**	
OCBI8	0.297	0.142	0.134	0.086	**0.676**	
OCBO1	0.366	0.017	0.053	0.117	**0.668**	
OCBO2	0.280	0.104	0.103	0.125	**0.756**	
OCBO3	0.230	0.124	−0.065	0.183	**0.729**	
OCBO4	0.135	0.179	0.018	0.176	**0.786**	
OCBO5	0.110	0.173	0.085	−0.030	**0.809**	
OCBO6	0.116	0.198	0.040	0.158	**0.759**	
OCBO7	0.183	0.189	0.098	0.109	**0.785**	
OCBO8	0.107	0.187	0.058	0.102	**0.719**	
Eigenvalues	11.379	5.170	5.083	4.012	3.372	
Variance explained (%)	26.463	12.022	11.821	9.330	7.843	
Accumulative variance explained (%)	26.463	38.485	50.306	59.636	67.478	

Abbreviation: OE = Overall evaluation, PS = Political skill, NS = Need for status, TV = Task visibility, OCB = Organizational citizenship behavior.

**Table 2:** Descriptive statistics and correlation analysis between variables.

Variable	Mean	S.D.	1	2	3	4	5	6	7	8
1. Age	35.24	6.98								
2. Gender	0.49	0.50	0.05							
3. Tenure	5.89	5.23	0.50 ***	0.04						
4. Education	2.81	0.62	−0.22 ***	0.01	−0.10					
5. Need for Status	4.72	0.90	−0.14 *	−0.03	0.05	0.18 **				
6. Political Skill	4.73	0.86	−0.08	−0.21 ***	0.10	0.22 ***	0.49 ***			
7. Task Visibility	4.55	0.61	0.09	−0.03	0.06	−0.01	0.11	0.24 ***		
8. OCB	5.01	0.85	−0.05	−0.14 *	0.13 *	0.25 ***	0.32 ***	0.35 ***	0.23 ***	
9. Overall Evaluation	4.98	1.17	0.04	−0.12 *	0.17 **	0.16 **	0.20 **	0.14 *	0.06	0.76 ***

N = 299, *: *p* < 0.05, **: *p* < 0.01, ***: *p* < 0.001. Two-tailed test.

**Table 3:** Confirmatory factor analysis.

Model	No. of Factors	χ^2^	df	Δχ^2^	RMSEA	CFI	IFI
Baseline model	Five factors: NS, PS, TV, OCB, OE	1951.71	821		0.07	0.90	0.90
Model 1	Four factors: (NS + PS), TV, OCB, OE	2640.94	825	289.23 **	0.09	0.84	0.84
Model 2	Four factors: NS, PS, TV, (OCB + OE)	2612.89	825	661.18 ***	0.09	0.85	0.85
Model 3	Three factors: (NS + PS + TV), OCB, OE	3397.10	828	1445.39 ***	0.10	0.77	0.78
Model 4	Two factors: (NS + PS + TV), (OCB + OE)	4050.36	830	2098.65 ***	0.11	0.72	0.72

Note: ** *p* < 0.01, *** *p* < 0.001. NS = Need for status, PS = Political skill, TV = Task visibility, OCB = Organizational citizenship behavior, OE = Overall evaluation; RMSEA = root mean square error of approximation; CFI = Comparative fit index, IFI = Incremental fit index.

**Table 4:** Hierarchical regression analysis results.

Variable	OCB			Overall Evaluation			
	Model 1	Model 2	Model 3	Model 4	Model 5	Model 6	Model 7
Control variables							
Age	−0.04	−0.06	−0.06	0.01	0.01	−0.01	0.05
Gender	−0.14 **	−0.11 *	−0.10	−0.12 *	−0.12 *	−0.10	−0.02
Tenure	0.16 **	0.15 *	0.11	0.18 **	0.18 *	0.14 *	0.05
Education	0.21 ***	0.19 ***	0.18 ***	0.16 **	0.16 **	0.16 **	0.01
Main effect							
Need for status (NS)	0.26 ***	0.18 **	0.12	0.16 **	0.16 **	0.07	−0.03
Moderating variables							
Political skill (PS)		0.13 *	0.17 **		−0.03	0.01	−0.11
Task visibility (TV)		0.17 **	0.19 ***		0.04	0.05	−0.10
Interaction effects							
NS ∗ PS			−0.18 **			−0.14 *	0.01
NS ∗ TV			00.17 **			0.24 ***	0.10
Mediating variable							
OCB							0.80 ***
R square	0.16	0.20	0.25	0.07	0.08	0.13	0.61
R square change		0.04 ***	0.05 ***		0.01	0.05 ***	0.48 ***

Note. N = 299,* *p* < 0.05, ** *p* < 0.01, *** *p* < 0.001.

**Table 5:** Bootstrapping results.

Independent Variable	Mediator	Dependent Variable	Moderator	Moderator Level	Conditional Indirect Effect	Product of Coefficients			Bootstrapping Bias-Corrected 95% Confidence Interval	
						SE	z	*p*	Lower	Upper
Need for status	OCB	Overall evaluation	Political skill	Lo (Mean − 1SD)	**0.292**	**0.111**	**2.63**	**<0.01**	0.070	0.495
				Mean	**0.193**	**0.072**	**2.68**	**<0.01**	0.051	0.331
				Hi (Mean + 1SD)	0.094	0.078	1.21	*ns*.	−0.045	0.258
			Task visibility	Lo (Mean − 1SD)	0.190	0.098	1.94	*ns*.	−0.010	0.380
				Mean	**0.227**	**0.066**	**3.44**	**<0.01**	0.091	0.347
				Hi (Mean + 1SD)	**0.263**	**0.073**	**3.60**	**<0.01**	0.131	0.409

Note. Bootstrap sample size = 1000. Coefficients in bold indicate significant mediation.

## Data Availability

Data is unavailable due to privacy or ethical restrictions.
